# Cross-Sectional Mediation Analysis: How Registered Nurses’ Knowledge, Attitude, and Practice Influence Adherence to Pressure Injury Prevention

**DOI:** 10.3390/healthcare14121760

**Published:** 2026-06-18

**Authors:** Regie Buenafe Tumala, Mousa Yahya Asiri, Sahar Abdulkareem Alghareeb

**Affiliations:** 1 Medical-Surgical Nursing Department, College of Nursing, King Saud University, Riyadh 12372, Saudi Arabia; rtumala@ksu.edu.sa; 2 Doctor of Philosophy in Nursing Program, College of Nursing, King Saud University, Riyadh 12372, Saudi Arabia; 3 Fundamentals of Nursing Department, College of Nursing, Imam Abdulrahman Bin Faisal University, Dammam 31441, Saudi Arabia; saalghareb@iau.edu.sa

**Keywords:** adherence, attitude, knowledge, practice, pressure injury prevention, nurse, mediation analysis

## Abstract

**Background:** Pressure injuries (PIs) remain a major and escalating global patient safety concern, affecting 12.8% of hospitalized patients worldwide and contributing to rising prevalence and mortality rates from 5.63 to 8.18 and 0.31 to 0.47 per 100,000 population, respectively. The economic burden of PIs is substantial, amounting to $26.8 billion annually in the United States and $9608 per patient in the Kingdom of Saudi Arabia (KSA). However, adherence toward pressure injury prevention (PIP) guidelines among registered nurses (RNs) remains critically inconsistent. **Objectives:** The aim of this study was to examine whether attitude and practice function as parallel mediators in the relationship between knowledge and adherence to PIP guidelines among RNs, adjusting for age and years of experience. This aim was addressed through three-fold objectives: to assess RNs’ knowledge, attitude, practice (KAP) and adherence to PIP guidelines, evaluate the direct knowledge–adherence relationship, and quantify the two mediated pathways. **Methods:** A cross-sectional mediation study recruited 166 RNs from 52 clinical units at Prince Sultan Military Medical City in Riyadh, KSA, using convenience sampling with data collected from 5 to 15 March 2026. Validated instruments assessed KAP and adherence to PIP guidelines. Pearson correlations, multiple linear regression, and parallel mediation (Hayes PROCESS macro, model 4; 5000 bootstrap resamples) were performed with age and years of experience as covariates. **Results:** Mean scores indicated low knowledge (7.31/25; 29.2%), negative attitude (28.20/52; 54.2%), poor practice (36.10/110; 32.8%), and low adherence (28.40/90; 31.6%). Regression explained 36.8% of the variance in adherence to PIP guidelines (Adjusted R^2^ = 0.35), with knowledge (β = 0.22; *p* < 0.003), attitude (β = 0.30; *p* < 0.001), practice (β = 0.20; *p* = 0.006), and years of experience (β = 0.12; *p* = 0.04) emerging as significant predictors. Both attitude (unstandardized indirect effect = 0.40; 95% Boot CI [0.20, 0.64]) and practice (indirect effect = 0.30; [0.10, 0.60]) significantly mediated the knowledge–adherence relationship, while knowledge retained a significant direct effect (B = 0.70, *p* = 0.003), indicating partial mediation. **Conclusions:** This study was the first to employ a parallel mediation analysis to examine KAP as concurrent predictors of adherence toward PIP guidelines within a tertiary military healthcare setting in the KSA. The mediating roles of attitude and practice, together with the direct effect of knowledge, indicated that adherence to PIP guidelines is shaped by interconnected cognitive and behavioral mechanisms. Persistently low KAP levels and low adherence, along with the predictive influence of all KAP domains and RNs’ years of experience, underscored the urgent need for integrated interventions that strengthen KAP to improve adherence and prevent PIs.

## 1. Introduction

Pressure injuries (PIs) are localized damage to the skin and underlying soft tissue, typically occurring over bony prominences as a result of sustained mechanical loads from pressure, shear, or friction [1]. PIs represent a significant and widespread patient safety challenge in contemporary healthcare systems globally [2]. In addition, PIs correlate with elevated morbidity, prolonged hospitalization, and greater healthcare costs [3], contributing to rising prevalence and mortality rates from 5.63 to 8.18 and 0.31 to 0.47 per 100,000 population, respectively [4]. Nutritional status plays an important role in both the prevention and healing of PIs [5]. Evidence suggests that inadequate nutrition and lifestyle factors increase susceptibility, while nutritional supplementation enriched with protein and amino acids supports tissue repair and wound healing [5]. These findings highlight the importance of integrating nutritional considerations into pressure injury prevention (PIP) strategies [5]. PIs have a substantial epidemiological impact worldwide and are studied extensively [3]. However, this discussion posits associations among knowledge, attitudes, and practices (KAP), and adherence without grounding these claims in the theoretical frameworks that substantiate them, including the theory of planned behavior, the classic KAP model, and empirical nursing studies that independently validate each of the KAP and adherence linkages.

A pivotal systematic and meta-analysis conducted by Li et al. [6], aggregating data from 2,579,049 hospitalized adults across 39 studies, determined a pooled global prevalence of 12.8% and a hospital-acquired pressure injury (HAPI) rate of 8.4%, equating to an incidence density of 5.4 per 10,000 patient days [6]. Geographical differences in prevalence further complicate the global landscape [6]. In a systematic assessment in Europe, Moore et al. [7] found a median acute care prevalence of 10.8%, with country-specific estimates varying from 4.6% to 27.2%. In the United States—in what remains the most extensive national economic analysis to date—a Markov simulation was used to model the expenses of all stages of HAPIs from the hospital perspective [8]. It estimated that the yearly national expenditure for HAPI care may exceed $26.8 billion [8]. In Saudi Arabia, Asiri et al. [9] reported a total cost of $9608 per patient associated with PIs, equating to an estimated $34.42 per day per patient [8]. Additionally, earlier research conducted in Saudi Arabia, a well-studied Arab healthcare context, revealed that PI rate in the country’s intensive care units (ICUs) exceeds 26% [10].

Registered nurses (RNs) are integral to PIP through risk assessment, repositioning, skin surveillance, moisture management, and support surface selection [11]. Empirically, knowledge has been identified as a prerequisite for competent PIP decision-making, yet knowledge alone does not determine behavioral outcomes [12]. Attitudes towards PIP, encompassing perceived responsibility, professional value and outcome expectancy, shape the motivational commitment that converts knowledge into preventive intent [13]. In turn, consistent practice operationalizes the intent into observable clinical behaviors and, ultimately, guideline adherence [14]. Accordingly, in the present study, attitude and practice are theoretically and empirically positioned as mediating factors rather than merely co-occurring variables in the knowledge–adherence pathway.

In the most comprehensive synthesis to date, Asiri et al. [13] conducted a systematic review and meta-analysis, aggregating data from studies across multiple countries. The authors identified that the most critical methodological limitation in this field is the lack of studies formally modeling KAP variables as collective, concurrent predictors of PIP adherence, and they called explicitly for regression-based quantitative designs to be conducted [13]. The present study was specifically developed to respond to this call [13]. Although growing research attention is being paid to PIP, evidence remains limited regarding how RNs’ knowledge interacts with other determinants to influence adherence to recommended preventive measures, particularly with respect to the mediating roles of attitude and practice. Understanding the relationships between RNs’ KAP and adherence in relation to PIP guidelines is essential for designing effective strategies to improve preventive care in clinical settings.

**Figure 1:**
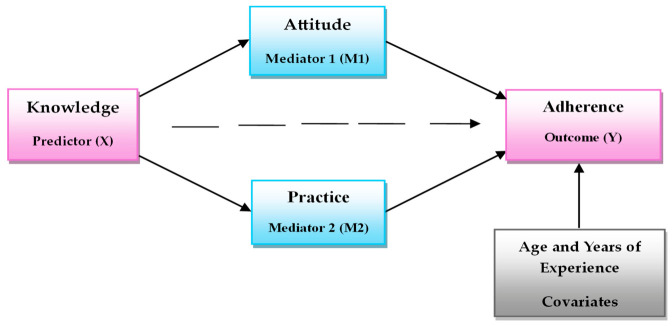
Conceptual Model Illustrating Registered Nurses’ Attitude and Practice as Parallel Mediators between the Relationship of Knowledge and Adherence to Pressure Injury Prevention Guidelines.

Thus, this study aimed to examine the mediating roles of attitude and practice in the relationship between RNs’ knowledge and adherence to PIP, adjusting for age and years of experience. Specifically, the study assessed the levels of KAP and adherence relating to PIP among RNs in a tertiary military hospital in Saudi Arabia. In addition, the study evaluated the direct effect of knowledge on adherence to PIP and determined the extent to which attitude and practice mediate the relationship between knowledge and adherence to PIP recommendations or guidelines among RNs, while controlling age and years of experience. Accordingly, a conceptual model (see Figure 1) was developed to visually represent the anticipated parallel mediation pathways and to situate the analysis within a clearly defined theoretical framework. The model reflected the empirical relationships confirmed in the mediation analysis conducted in the study. Knowledge functioned as the core cognitive determinant, while attitude and practice operated as parallel mediators linking the relationship between knowledge and adherence to PIP guidelines, consistent with both KAP framework and the study’s mediation results. Age and years of experience contributed as covariates, supporting their inclusion in the model. The model accurately represents the pathways validated in the study and aligns with the study’s aim, objectives, research questions, hypotheses, and analytic design. Furthermore, a set of research questions and hypotheses was articulated to translate the study objectives into testable propositions. Together, these components establish a coherent analytic structure for examining how RNs’ KAP interact to shape adherence to PIP guidelines, thereby aligning the empirical approach with the study’s theoretical foundations.

### 1.1. Research Questions

1. What are the levels of KAP and adherence to PIP guidelines among RNs?
2. To what extent do KAP independently predict adherence to PIP guidelines when controlling for age and years of experience?
3. Do attitude and practice mediate the relationship between knowledge and adherence to PIP guidelines within the proposed parallel mediation model?

### 1.2. Hypotheses

1. Knowledge level is positively and directly associated with adherence to PIP guidelines.
2. Attitude significantly mediates the relationship between knowledge and adherence to PIP guidelines.
3. Practice significantly mediates the relationship between knowledge and adherence to PIP guidelines.
4. Attitude and practice function as parallel mediators, yielding a statistically significant total indirect effect.
5. Age and years of experience independently predict adherence to PIP guidelines.

## 2. Materials and Methods

### 2.1. Research Design

A cross-sectional, correlational design utilizing mediation analysis was implemented in this study. This design facilitates the concurrent evaluation of variables at a specific moment, making it appropriate for investigating the relationships among the independent predictor (knowledge); the mediating variables (attitude and practice); and the outcome variable (adherence to PIP) [15]. The research was conducted and documented in adherence to the Strengthening the Reporting of Observational Studies in Epidemiology (STROBE) guidelines (Supplementary Materials) [16], thereby ensuring methodological rigor and transparency in reporting.

### 2.2. Study Setting

The study was conducted at Prince Sultan Military Medical City (PSMMC) in Riyadh, Saudi Arabia, which is a tertiary military hospital with a capacity of approximately 1279 beds and offers extensive acute and critical health services. Data collection took place across 52 clinical units, encompassing different units and other clinical settings where PI occurrences are frequently documented. These units were selected because they reflect a patient population characterized by immobility, complex comorbidities, and prolonged hospitalization, all of which elevate PI risk. PSMMC was chosen as the study site due to its substantial bed capacity, advanced clinical services, and its role as a major referral center serving diverse patient demographics, thereby providing a representative environment for examining adherence to PIP guidelines.

### 2.3. Participants

This study included RNs engaged in direct bedside patient care with a minimum of one year of clinical experience. This criterion was implemented to ensure that participants had adequate clinical exposure to actively interact with KAP and adherence to PIP guidelines. RNs in their first year at PSMMC were undergoing a structured orientation and preceptorship period, during which practice is in an early developmental stage and may not reflect independent clinical judgement. RNs were also excluded if they held administrative or non-clinical positions during the study’s data-collection period, or if they were absent from work because of leave or illness for the entire survey duration.

### 2.4. Sample Size

The required sample size was calculated utilizing G*Power software version 3.1.9.7 [17]. With a medium effect size (*f*^2^ = 0.15), a significance level of 0.05, a statistical power of 0.95, and five predictors (KAP, age, and years of experience), the minimum required sample size was calculated to be 138 participants. To account for an anticipated 20% non-response rate and to maintain adequate statistical power for the planned statistical analyses [17], the target sample size was increased to 166 RNs.

### 2.5. Sampling Technique

A convenience sampling method was used to recruit RNs from selected units within the study environment at PSMMC. This strategy, while potentially introducing selection bias, was deemed suitable because of the practical constraints of recruiting participants in a busy clinical setting [15]. To enhance sample representativeness, all qualified RNs in the designated units were approached sequentially during their scheduled working hours throughout the data collection phase. No RNs declined participation or failed to submit a completed survey, resulting in a final response rate of 100% [15].

### 2.6. Research Instruments

The survey included five sections. The first was a sociodemographic questionnaire developed by the authors to collect RNs’ information, including gender, age, ethnicity, educational qualification, years of experience, clinical unit, PIP education or training, competency in PIP, and last reading of PIP guidelines. All other instruments, as presented below, were obtained with the primary authors’ or copyright holders’ permission.

The second section assessed RNs’ knowledge using the Pressure Ulcer Knowledge Assessment Tool (PUKAT) version 2.0, developed by Manderlier et al. [18]. The PUKAT 2.0 is a validated multiple-choice instrument comprising 25 items across six domains: etiology, classification and observation, risk assessment, nutrition, preventive interventions, and specific patient groups [18]. Items are scored 0 or 1, with higher total percentages indicating greater theoretical knowledge [18]. In the present study, the total PUKAT score was converted to a percentage of the maximum possible score and classified into three knowledge levels: high knowledge (≥80%, ≥20 points), moderate knowledge (60–79%, 15–19 points), and low knowledge (<60%, <15 points). The tool has acceptable reliability and validity; it showed an intraclass correlation coefficient of 0.69 and a content validity index (CVI) of 0.78 [18].

The third section of the questionnaire assessed RNs’ attitude using the Attitude towards Pressure Ulcer Prevention (APuP) instrument developed by Beeckman et al. [19]. The APuP measures nurses’ attitudes towards PIP, including their perceptions, motivation, and perceived responsibility for PI preventive care. The instrument comprises 13 items rated on a four-point Likert scale ranging from 1 (strongly disagree) to 4 (strongly agree) [19]. Several negatively worded items are reverse-scored, such that higher total scores reflect more positive attitude toward PIP [19]. In the current study, the total APuP score was converted to a percentage of the maximum possible score and categorized into three attitude levels: positive attitude (≥80%, ≥42 points), moderate attitude (60–79%, 31–41 points), and negative attitude (<60%, <31 points). Previous validation study reported acceptable content validity and satisfactory internal consistency for the APuP [19]. The scale demonstrated good internal reliability, with a Cronbach’s alpha of 0.79 and CVI of 0.87 [19].

The fourth section assessed RNs’ PI preventive practices using a standardized practice questionnaire adapted from Thomas and Nain [20]. The instrument consists of 22 items measuring RNs’ practices in relation to PIP [20]. Each item is rated on a five-point Likert scale ranging from 1 (never) to 5 (always) [20]. The total practice score was calculated by summing the item responses, with higher scores indicating better adherence to evidence-based PIP practices [20]. In the present study, the total practice score was converted to a percentage of the maximum possible score and grouped into three practice levels: good practice (≥80%, ≥88 points), moderate practice (60–79%, 66–87 points), and poor practice (<60%, <66 points). The instrument demonstrated good internal consistency in the prior study, with a Cronbach’s alpha coefficient of 0.89 and CVI of 0.79 [20].

The fifth section assessed adherence to guideline-based preventive recommendations using the Questionnaire to evaluate nurses’ Adherence to Recommendations for Preventing Pressure Ulcers (QARPPU), developed by Moya-Suárez et al. [21]. The validated instrument includes 18 items rated on a five-point Likert scale ranging from 1 (never) to 5 (always) [21]. Total scores range from 18 to 90, with higher scores indicating greater adherence to PIP recommendations. Specifically, the total adherence score was converted to a percentage of the maximum attainable score and subsequently grouped into three adherence levels: high adherence (≥80%, ≥72 points), moderate adherence (60–79%, 54–71 points), and low adherence (<60%, <54 points). The QARPPU demonstrated good psychometric properties, with a reported internal consistency of Cronbach’s alpha of 0.89 [21].

All four instruments were administered in their original English versions [18,19,20,21]. This was deemed appropriate given that the sample population in this study consisted predominantly of English-proficient expatriate nurses.

### 2.7. Data Collection

Data were collected from 5 to 15 March 2026 by self-administered surveys disseminated electronically via Google Forms and distributed on-site through a restricted link by unit-level supervisors and the research team [15]. The surveys were administered to the participating RNs in the designated clinical units, and were completed exclusively on-site with the help of unit-level supervisors during scheduled working hours across the 52 clinical units. The survey link was not posted on social media, mailing lists, or any public platform, thereby eliminating open self-selection from an undefined population [15]. Before completing the survey, the researchers explained the study objectives and protocol, the voluntary nature of participation, and confidentiality protections. Informed consent was obtained from all participants prior to initiating the online survey [15]. A member of the research team was physically present in the unit during survey completion to verify participant eligibility against the inclusion criteria, clarify item wording on request, and confirm that the instrument was completed in a single session without external consultation. Response quality safeguards were implemented [15]. The platform enforced mandatory responses for all scale items, preventing missing data; IP-based restriction limited one submission per device; and timestamp audits were conducted to identify and exclude implausibly rapid completions, although none were detected. Average completion time ranged from 20 to 30 min, and participants retained the option to decline participation or withdraw at any point prior to final submission without consequence; no refusals were recorded among the RNs who voluntarily and willingly completed the online survey.

### 2.8. Ethical Considerations

Ethical approvals for this study were secured from the Institutional Review Board (IRB) of King Saud University (Approval Number: KSU-HE-26-0192, dated 4 March 2026, with an initial approval date of 26 February 2026) and from the PSMMC Ethics Committee (Reference Number: E-2824, dated 16 February 2026). The research was performed in compliance with the ethical standards of the Declaration of Helsinki and relevant national research regulations [15]. Participation was voluntary, and written informed consent was obtained from all participants before data collection commenced. Participants were notified that they could refuse or withdraw from the study at any moment without repercussions [15]. No personally identifiable information was collected; all data were anonymized and stored securely on password-protected institutional systems accessible only to the study team [15].

### 2.9. Data Analysis

All statistical analyses were performed with IBM SPSS Statistics (Version 30.0) [22] and the Hayes PROCESS macro (Model 4) for SPSS [23]. Descriptive statistics were calculated for continuous research variables, including frequencies and percentages for the categorical variables, means, standard deviations, and ranges, were calculated for continuous research variables [22]. The normality of continuous variables was assessed by examining skewness and kurtosis values, with thresholds of ±2 for skewness and ±7 for kurtosis, which are typically considered indicative of approximate normality [22].

Pearson correlation coefficients were calculated to examine the bivariate associations between KAP variables and adherence to PIP guidelines. The assumptions for Pearson correlation were verified, including (1) interval- or ratio-level measurement, (2) near-linear relationships confirmed through scatterplot inspection, (3) absence of significant outliers, and (4) approximate normality of distributions [22]. A multiple linear regression model was then constructed with adherence to PIP guidelines as the dependent variable and KAP, age, and years of experience as predictors [24]. Regression diagnostics included evaluation of multicollinearity using tolerance values and variance inflation factors (VIFs); assessment of residual autocorrelation using the Durbin–Watson statistic; and inspection of residual histograms and quantile–quantile (Q–Q) plots [24].

Parallel mediation analysis was conducted using the Hayes PROCESS macro (Model 4), with 5000 bootstrap samples and a 95% percentile confidence interval (CI) [23]. In this model, knowledge was specified as the predictor variable (X), adherence as the outcome variable (Y), and attitude and practice as concurrent mediators (M1 and M2, respectively) [23]. Age and years of experience were included as covariates (see Figure 1). Indirect effects were considered statistically significant when the 95% bootstrap CI did not include zero [23]. Statistical significance for all other analyses was set at *p* = 0.05. No subgroup or interaction analyses were planned or conducted. The study was powered specifically for the primary parallel mediation model (N = 166), and the available sample size was insufficient to support reliable stratified analyses across clinical unit type, nationality, or educational level. Exploratory subgroup analyses were therefore deferred to future multisite studies.

## 3. Results

**Figure 2:**
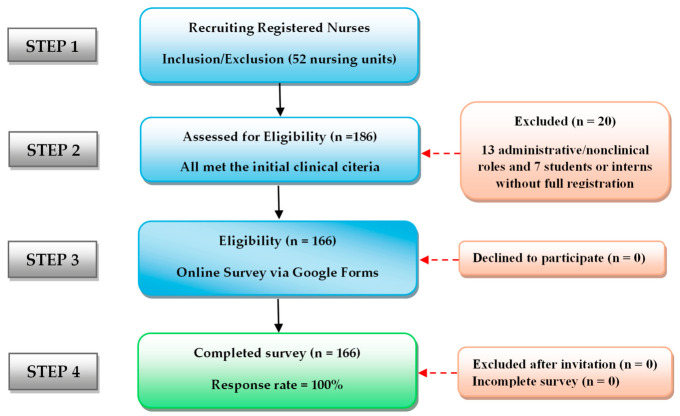
Recruitment Flow Diagram.

A total of 186 RNs were assessed for eligibility and approached sequentially. Of these, 20 were excluded based on predefined criteria (13 were in administrative or other non-clinical roles; 7 held student or intern status), resulting in 166 eligible participants. All eligible nurses provided informed consent, participated in the study, and completed the survey, yielding a 100% response rate within the eligible sample. No refusals, withdrawals, or incomplete responses were recorded. All participants completed every survey item, resulting in no missing data (see Figure 2).

### 3.1. Demographic and Professional Characteristics of Participants

A total of 166 RNs participated in this study, with no missing data across any variable. The sample was predominantly female (n = 137, 82.5%), whereas male nurses comprised 17.5% (n = 29). In relation to ethnicity, Filipino nurses formed the largest subgroup (n = 76, 45.8%), followed by Indian nurses (n = 65, 39.2%), with smaller proportions of Arab nurses (n = 13, 7.8%) and nurses of other nationalities (n = 12, 7.2%). Most of the participants held a bachelor’s degree (n = 98, 59.0%), followed by diploma-level qualifications (n = 36, 21.7%) and master’s degrees (n = 32, 19.3%). Almost half of the participants (n = 79, 47.6%) worked in critical care settings such as ICUs and critical wards, and the remaining 52.4% (n = 87) were deployed in acute care areas, including the emergency department (ED) and medical wards. A substantial majority (n = 155, 93.4%) reported never having received formal education or training on PIs, yet 89.8% (n = 149) indicated that they possessed PI-related competency. More than half of the participants (n = 96, 57.8%) had read PI guidelines within the past year, while 42.2% (n = 70) reviewed them one to two years earlier. Participant demographic and professional characteristics are summarized in Table 1.

### 3.2. Descriptive Statistics for Continuous Variables Included in the Parallel Mediation Model

Descriptive statistics for all continuous study variables are presented in Table 2. The mean knowledge score was 7.31 (SD = 3.61, range: 2–16), indicating low PI-related knowledge among RNs. The mean attitude score was 28.20 (SD = 5.20, range: 13–36), reflecting a generally positive attitude towards PIP practices. Practice scores averaged 7.01 (SD = 3.01, range: 1–12). Adherence, the primary outcome variable, had a mean of 28.40 (SD = 11.8, range: 18–75). The mean age of RNs was 36.80 years (SD = 7.71), and the mean years of professional experience score was 11.72 (SD = 5.60).

Inspection of skewness and kurtosis values indicated that knowledge (0.40/−0.61); attitude (−0.61/0.11); practice (−0.13/−0.83); age (0.65/0.33); and experience (0.32/−0.8) were all within acceptable bounds for normality (skewness < 2; kurtosis < 7). The overall adherence variable exhibited moderate positive skewness (1.80) and a kurtosis value of 3.61, indicating a slight departure from normality. However, this deviation was deemed acceptable for regression-based analyses given the adequate sample size (N = 166), which meets the assumptions of the central limit theorem assumptions. Collinearity diagnostics in the subsequent regression analysis confirmed the absence of multicollinearity (all VIF < 2; all tolerance > 0.6).

### 3.3. Correlational Analysis

Pearson product–moment correlations were computed to examine associations between KAP and adherence scores. As presented in Table 3, all intervariable correlations were statistically significant at the *p* < 0.001 level. Knowledge demonstrated moderate positive associations with attitude (*r* = 0.49); practice (*r* = 0.58); and overall adherence (*r* = 0.48). Attitude was significantly associated with practice (*r* = 0.51) and overall adherence (*r* = 0.51). Practice was similarly correlated significantly with overall adherence (*r* = 0.48). These findings indicate that higher knowledge level was associated with more favorable attitude and better PIP practice, and that each KAP domain was significantly correlated with adherence to PIP guidelines. The magnitude and positive direction of these associations provided a strong conceptual foundation for the subsequent regression and mediation analyses.

### 3.4. Multiple Linear Regression Analysis

A simultaneous multiple linear regression was conducted to examine the independent contributions of KAP, age, and years of experience to overall adherence. Prior to analysis, the assumptions of multiple regression were evaluated. Inspection of the Durbin–Watson statistic (DW = 1.9) indicated independence of residuals. The normal Q–Q plot and the histogram of standardized residuals suggested approximate normality of errors, while the scatterplot of standardized residuals against standardized predicted values showed no discernible pattern, supporting homoscedasticity. No evidence of multicollinearity was detected (all VIF < 2; all tolerance > 0.6). The regression model was statistically significant, *F*(5, 160) = 24.5, *p* < 0.001, and explained a substantial proportion (36.8%) of variance in adherence (*R*^2^ = 0.40, adjusted *R*^2^ = 0.35).

Attitude emerged as the strongest significant predictor of adherence (*B* = 0.70, β = 0.30, *t* = 4.40, *p* < 0.001, 95% CI [0.38, 0.98]), indicating that a one-unit increase in attitude score was associated with a 0.70-unit increase in adherence, controlling for other variables. Practice was the next most influential predictor (*B* = 0.8, β = 0.2, *t* = 2.8, *p* = 0.006, 95% CI [0.20, 1.41]); followed by knowledge (*B* = 0.70, β = 0.22, *t* = 3.10, *p* = 0.003, 95% CI [0.25, 1.2]). Years of experience also showed a modest but significant positive association with adherence (*B* = 0.30, β = 0.12, *t* = 2.1, *p* = 0.04, 95% CI [0.01, 0.5]), suggesting that RNs with more years of experience had higher levels of PIP adherence. Age did not independently predict adherence (*B* = −0.20, β = −0.08, *t* = −1.6, *p* = 0.12). Full model estimates are presented in Table 4.

### 3.5. Mediation Analysis

To investigate the mechanisms through which knowledge influences overall PIP adherence, a parallel multiple mediation analysis was conducted using the Hayes PROCESS macro (Version 4) for SPSS (model 4) [19], with attitude and practice as simultaneous mediators and age and years of experience as covariates. Bias-corrected CIs were estimated using 5000 bootstrap samples to enhance the robustness of indirect effect estimation. The results are summarized in Table 5.

#### 3.5.1. Path Coefficients

Knowledge significantly predicted attitude (Path a1: *B* = 0.61, *t* = 6.30, *p* < 0.001, 95% CI [0.41, 0.80]), indicating that higher knowledge was associated with more favorable PIP attitudes. Knowledge also significantly predicted practice (Path a2: *B* = 0.41, *t* = 7.91, *p* < 0.001, 95% CI [0.30, 0.51]), suggesting that greater PIP knowledge was associated with stronger PIP practices. In the outcome model, both attitude (Path b1: *B* = 0.70, *t* = 4.40, *p* < 0.001) and practice (Path b2: *B* = 0.81, *t* = 2.80, *p* = 0.006) emerged as significant predictors of adherence after adjustment for covariates. The direct effect of knowledge on adherence, controlling for both mediators, remained statistically significant (c’ = 0.70, *t* = 3.10, *p* = 0.003, 95% CI [0.25, 1.21]), indicating partial mediation.

#### 3.5.2. Indirect Effects

The indirect effect of knowledge on overall adherence through attitude was significant (indirect effect = 0.41, Boot SE = 0.10, 95% Boot CI [0.20, 0.64]), as the CI excluded zero. Similarly, the indirect effect via practice was significant (indirect effect = 0.3, Boot SE = 0.12, 95% Boot CI [0.098, 0.60]), providing further evidence of substantial mediation. The total indirect effect across both mediators was 0.70 (Boot SE = 0.17, 95% Boot CI [0.43, 1.11]), further confirming strong mediation. Together, these findings supported a partial mediation model, whereby knowledge exerts both direct and indirect effects on adherence to PIP guidelines, with attitude and practice operating as significant parallel mediators. Accordingly, the total effect of knowledge on adherence was apportioned into a direct effect (c′ = 0.70) and two significant indirect pathways, each contributing meaningfully to RNs’ PIP adherence.

## 4. Discussion

### 4.1. Key Findings

The findings of the present study have direct policy relevance to the transformative healthcare agenda of Saudi Vision 2030 [25]. The Vision’s healthcare transformation program explicitly commits to elevating patient safety standards; strengthening clinical workforce competencies; and achieving measurable improvements in healthcare quality indicators across Ministry of Health and military medical facilities [25]. Within this policy context, examining how knowledge influences adherence to PIP guidelines through a mediation analysis involving attitude and practice becomes particularly important. Asiri et al.’s study [13] found lack a formal mediation analysis in which KAP are modelled as concurrent, structured predictors of adherence to PIP guidelines; however, the parallel mediation analysis conducted in the present study indicated that both attitude and practice showed significant indirect effects of knowledge on adherence to PIP guidelines. Additionally, knowledge maintained a significant direct effect on adherence after controlling both mediators. The high PIP certification rate (89.83%) among RNs in this study may likely reflect contractual or institutional compliance requirements rather than serving as a robust indicator of clinical proficiency or excellence in adhering to PIP guidelines. The PIP guideline-reading data may reveal that knowledge currency was inconsistent across the nursing workforce, aligning with the approach used by Zhu et al. [26]. However, caution is warranted in interpreting this finding, as PIP certification or guideline-reading was not included in the mediation model and therefore cannot be assumed to function as mediating or explanatory factors.

Together, these findings explain the observed strong relationship between KAP and adherence to PIP guidelines. That is, RNs may know the guidelines exist and may have signed off on competency, but actual bedside adherence requires sustained education, regular refresher courses, direct supervision, and institutional accountability structures that go beyond contract renewal alone. Attitude was identified as the greatest independent predictor of adherence, followed by knowledge and then practice. The model accounted for 36.8% of the variance in adherence. RNs’ years of clinical experience independently predicted adherence, but age did not, highlighting the importance of practice accumulation in nursing practice. This educational discrepancy represents the greatest empirical indicator within the dataset. In other words, the study’s most important finding is structural: knowledge does not lead to adherence through a single unmediated pathway but operates through a sequential behavioral chain, with attitudinal orientation and clinical practice habits each contributing a significant share of the effect. These findings have direct policy relevance within Saudi Vision 2030 and are discussed below in relation to the international KAP literature, the structural paradox posed by competency certification in the absence of formal education, and the implications for nursing practice and institutional policy.

### 4.2. Registered Nurses’ Knowledge of Pressure Injury Prevention

The mean PUKAT 2.0 score of 7.31 out of 25 observed in the present study was low in relation to international benchmarks [21]. In the original PUKAT 2.0 tool assessment validation study in Belgium, De Meyer et al. [27] reported a mean score of approximately 50.7%, more than double that found in the present study, reflecting the sustained investment in PIP education characteristic of nurses and nursing assistants in that context. In a study conducted in Sweden, Källman and Suserud [28] found that most nurses indicated adequate foundational knowledge, with persistent gaps only in risk assessment and nutrition, domains in which the present study’s sample also performed poorly. A broader synthesis by Asiri et al. [13] confirmed that knowledge deficits are systematically more pronounced in Middle Eastern and Asian settings, a pattern attributable to structural differences in nursing education, continuing professional development, and the integration of wound care into undergraduate training. In China, Li et al. [29] conducted a multicenter latent profile analysis of 2409 nurses and identified three distinct KAP profiles: 12.82% fell into the low-KAP cluster, 52.32% into the moderate-KAP cluster, and 34.95% into the high-KAP cluster. The total KAP score of the participants reported across these clinical settings was 63.44 ± 7.69 [29]. Similarly, Luo et al. [30], studying hospitals in China’s Shaanxi Province, reported a mean knowledge score of 61.56%, with the lowest scores in wound knowledge at 56.7% [30], a pattern consistent with the present findings. In Middle Eastern and African contexts, Nuru et al. [31] documented that only 45.6–54.4% of Ethiopian nurses had adequate PIP knowledge, with the weakest knowledge being about prevention of PI [31]. The nurses had poor knowledge on three domains: risk assessment, skin care, and management for mechanical loads [31]. ALfadhalah et al. [32] reported that the median score of nurses’ knowledge on preventing PIs was 73.2%, and in the present study, only 29.2% of the knowledge test items were answered correctly by 90% of participants or more of the participants. In Australia, a study examining 300 nurses in a tertiary hospital by Fulbrook et al. [33] found that only 65% of the nurses indicated comprehensive PIP knowledge, indicating persistent bedside knowledge deficits even in well-resourced healthcare systems [33]. On the other hand, Demarré et al. [34] reported that Belgian RNs scored only 29.3% for PIP knowledge. Taken together, the present findings provide further evidence of a critical worldwide PIP knowledge deficit and align with results documented in most European and Australian studies [27,28,33,34]. These patterns underscore the urgency of formalizing PIP education at the current study’s setting in PSMMC as a patient safety imperative rather than an optional provision. Collectively, the existing empirical evidence confirms that although PI knowledge deficits are global, their severity is moderated by region, institutional type, and the degree of formalization of PI education [13,35].

### 4.3. Registered Nurses’ Attitudes Toward Pressure Injury Prevention

The mean APuP score of 28.2 out of 52 in this study signifies generally negative attitude to PIP, inconsistent with the international APuP literature [36]. Beeckman et al. [36] reported significant correlations with the knowledge and attitude of RNs, with odds of adherence being 3.07 times higher among nurses in Belgian acute care settings. Demarré et al. [34] reported that a more positive attitude was a significant predictor of adherence to PIP guidelines in care provision to clients at risk of PIs in nursing homes. These findings closely align with the present results despite substantial contextual differences [34,35,36]. This cross-continental convergence supports the interpretation that positive attitudes towards PIP reflect a feature of professional nursing identity rather than a product of country-specific supplies or education [34,35,36]. In Malaysia, Thomas and Nain [20] found that positive attitude coexisted with significant knowledge and practice deficits, with attitude exhibiting a weaker correlation with preventive behaviors than with knowledge. In their study of Chinese neonatal ICU nurses, Shi et al. [37] reported acceptable APuP scores, with inter-unit variation favoring nurses who had received PI-specific training. In the present study, the near absence of formal PIP education did not highly depress attitudinal scores to the same extent that it suppressed knowledge and adherence, supporting the argument that professional socialization sustains attitudinal orientation even in the absence of substantive knowledge. However, this interpretation should be approached cautiously, as formal PIP education was not included in the mediation analysis and therefore its potential explanatory role cannot be evaluated.

### 4.4. Registered Nurses’ Practice in Pressure Injury Prevention

The present study recorded an exceptionally low practice score (7.31/110), indicating a substantial gap between negative attitude and actual preventive behaviors, an imbalance that aligns with Thomas and Nain’s observation that unfavorable attitudes do not reliably translate into competent PIP practice. In contrast, Thomas and Nain [20] reported moderate practice scores in Malaysia despite comparable attitudinal profiles, attributing this discrepancy to structural barriers such as time constraints, workload, and inadequate equipment [13]. In the research context of Ethiopia, Nuru et al. [31] found that 89.9% of nurses did not perform regular PI risk assessments, 55.2% did not adhere to repositioning schedules, and fewer than 12.9% consistently documented preventive activities—patterns that are captured within the present study’s practice instrument [20]. Although these data arise from a resource-limited context, the directional consistency with the present findings is notable. Given the superior resourcing of PSMMC, knowledge deficits rather than structural constraints represent the more plausible primary mechanism underlying practice gaps [13]. PIP practice emerged as the weakest domain in the present study, even among RNs with low knowledge level, a pattern consistent with Jiang et al.’s findings in mainland China [15,38], underscoring the need to prioritize PIP within educational interventions for nurses. When taken together, these cross-country findings may suggest that improving adherence to PIP guidelines requires more than addressing structural barriers; it demands targeted educational and behavioral interventions that explicitly bridge the persistent knowledge-to-practice gap.

### 4.5. Registered Nurses’ Adherence to Pressure Injury Prevention

In the present study, the mean QARPPU score of 28.40 out of 90 (31.6%) represents one of the lowest adherence rates documented in the PIP nursing literature. The QARPPU was validated by Moya-Suárez et al. [21] in a Spanish hospital setting, showed substantially higher adherence, reflecting a more formally educated workforce and stronger institutional accountability structures. In China, Shi et al. [37] reported moderate adherence among Chinese neonatal ICU nurses using a comparable instrument, a level higher than that observed in the present study yet demonstrating the same pattern in which positive attitude toward PIP coexist with incomplete guideline adherence. In a prior scoping review, Cordina et al. [39] documented that adherence to PIP interventions remained inconsistent even in institutions with formal PIP protocols, a finding directly relevant to the present study, which similarly observed that achieving PIP competency certification coexisted with persistently low adherence to PIP guidelines. Alshahrani et al. [11] identified repositioning frequency, nutritional assessment and skin inspection documentation as the most commonly observed PIP adherence gaps with critically ill patients, precisely the domains assessed by the QARPPU [21], thereby strengthening confidence in the QARPPU as a clinically meaningful adherence index for this population. Nevertheless, this interpretation should be viewed with caution, as PIP competency certification was not incorporated into the mediation analysis, and its potential explanatory contribution therefore remains undetermined from the context of the current study.

### 4.6. Knowledge, Attitude and Practices as Predictors of Adherence: Regression and Mediation Evidence

The finding that attitude was the most powerful predictor of PIP adherence is in line with Shi et al. [37], who also identified attitude as the principal determinant of evidence-based PIP adherence in comparative clinical practice. Accordingly, this finding aligns with prior studies in different countries and clinical specialties that further supports the theoretical framework of the KAP [13], in which attitudinal evaluation is the most direct antecedent of behavioral action. Beeckman et al. [36] observed that nurses scoring higher on attitudinal instruments were significantly more likely to implement evidence-based repositioning and skin care regimens, independent of their PIP knowledge scores, and this finding is directly mirrored in the mediation model developed in the present study. The independent contribution of PI knowledge to adherence, beyond its indirect effects, is consistent with the conclusions of Pancorbo–Hidalgo et al. [40], whose large-scale Spanish study of 740 nurses found that higher PI knowledge predicted the use of validated risk assessment tools, appropriate support surface protocols, and preventive documentation, all components of the QARPPU construct [21,40].

All three KAP domains contribute independently to adherence to PIP guidelines. The independent contribution of RNs years of experience to adherence is consistent with Fulbrook et al.’s [33] finding that experienced nurses reported significantly higher rates of preventive documentation and risk assessment completion, independent of educational level. Similarly, Fulbrook et al. [33] and Demarré et al. [34] identified years of experience as the strongest demographic predictor of PI-specific practice, suggesting that experiential learning accumulates competencies that formal education alone does not fully instill [33,34].

The non-significant finding for the effect of age, despite the significant findings for the effect of experience effects, is in line with the broader clinical expertise literature, which distinguishes the practical gains of deliberate clinical practice from those of biological aging [13]. The relevant differentiator is the content and quality of PI-specific continuing education, rather than credential level. This interpretation is supported by the present finding that 93.42% of participants had no formal PI education regardless of their qualification level, effectively eliminating educational level as a differentiating variable in this sample. However, this inference should be interpreted cautiously, as formal PIP education was not incorporated into the mediation analysis and its independent contribution therefore remains analytically unresolved [3]. The formal confirmation of mediation through both PIP attitude and practice is this study’s most structurally novel contribution. The finding that knowledge predicted attitude more strongly than practice at the a-path level, while practice predicted adherence more strongly than attitude at the b-path level, indicates a structural asymmetry: knowledge may consolidate in behavioral routines before fully restructuring the deeper evaluative affective orientations embedded in professional socialization.

### 4.7. Competency Without Education Contradiction in Comparative Perspective

Li et al. [29] identified a subgroup of Chinese nurses with high PIP knowledge but moderate practice and adherence, a profile that implicitly illustrates the mediating role of practice. That is, PIP knowledge is insufficient to drive adherence in the absence of strong practice habits [29]. The present mediation model provides the first formal statistical specification of the mechanism implied by Li et al. [29]. Shi et al. [37] found that nurses scoring highest on attitudinal instruments were most likely to translate knowledge into documented preventive behaviors, consistent with the significant attitude-to-adherence pathway identified in this study. Thomas and Nain [20] made the conceptual argument in their Malaysian dataset that attitude and practice function as bridging variables between theoretical knowledge and preventive behavior, and the present study provides the quantitative substantiation of that argument. Tayyib et al. [10,41] noted that Saudi ICU nursing staff attributed PI incidence partly to insufficient training, despite institutional protocols being in place [10,41].

The present study makes two main contributions to knowledge beyond prior literature. First, it provides the first formal parallel-mediation analysis of the KAP and adherence pathway in the Saudi Arabian context of PIP, responding directly to the call by Asiri et al. [13]. Second, the mediation pattern, with a significant direct effect of knowledge together with significant indirect effects through attitude and practice, partitions the total effect of knowledge into its constituent pathways, thereby progressing beyond the bivariate or simple regression models characteristic of existing KAP studies.

In summary, this cross-sectional mediation study that attitude and practice mediated the relationship between PIP knowledge and PIP adherence among RNs in a Saudi Arabian tertiary military hospital, with attitude emerging as the dominant predictor of adherence. Convergence of these findings with evidence from Australia [33,42], Belgium [27,34], China [12,29,30,37,38], Ethiopia [31], Malaysia [20], Spain [40], and Saudi Arabia [10,41], strengthens confidence in the directional conclusions. The existence of worldwide competency certification, with near-absent formal education and with KAP and adherence levels far below what those credentials imply, constitutes a patient-safety signal requiring urgent, evidence-informed institutional action. These results provide critical direction for nursing leadership, management, education and policymakers, with the acknowledgement that single-site cross-sectional evidence is appropriate for informing intervention design rather than justifying immediate system-wide change. The current competency certification model requires systematic re-evaluation [13]. The coexistence of high certification rates with low formal training and inadequate knowledge indicates a structural disconnect between credentialing process and actual clinical competence [13]. Evidence consistently demonstrates that only multifaceted interventions integrating structured learning, skills teaching, and ongoing audit achieve sustained improvements in preventive practice [13]. The independent influence of clinical experience on PIP adherence supports the creation of structured mentorship programs, particularly in locations with a predominantly expatriate workforce. Combining mentorship within cultural terms with reactive education may bridge the gap between theoretical knowledge and clinical performance, ultimately strengthening PIP adherence.

### 4.8. Limitations

Several study limitations merit acknowledgement. First, the cross-sectional study design precluded confirmation of causal relationships, meaning that mediation was interpreted in a statistical rather than causal sense. Second, all variables were assessed through self-report, introducing potential social desirability bias and likely yielding an upper-bound estimate of adherence, even though KAP domains and adherence were rated low. Third, the single-center design and short data-collection period limit generalizability to hospitals in other regions and across both government and private healthcare systems, given the specific institutional context and workforce composition. Fourth, unmeasured contextual factors involving staffing ratios, resource availability, and organizational culture may have confounded the observed associations. Fifth, no formal sensitivity analyses were conducted. The moderate positive skewness of the adherence variable (skewness = 1.8) was considered acceptable for parametric inference given the adequate sample size (N = 166), consistent with the central limit theorem assumptions. Sixth, differences in undergraduate nursing curricula, clinical training and professional development systems may influence nurses’ KAP related to PIP.

### 4.9. Future Directions

Future research should move beyond cross-sectional study designs and progress toward longitudinal, intervention-based studies with pre–post assessments and follow-up at 3–6 months to more robustly establish structural pathways between KAP domains and PIP adherence. Objective adherence measures, direct observational audits, and electronic health record reviews are essential to overcome the limitations of self-reported data. Multisite studies using multilevel modelling are needed to disentangle individual KAP effects from organizational factors, including staffing, resource availability, and institutional PIP culture. Comparative evaluations of different educational delivery models will further inform evidence-based training strategies. Future research should also examine context-specific moderators, such as nationality, contract conditions, PIP education or training, competency certification in PIP, and educational background, and incorporate qualitative methods to capture RNs’ experiences with competency-based education, enabling the development of targeted, culturally responsive interventions.

## 5. Conclusions

This study concluded that attitude and practice significantly mediated the relationship between knowledge and adherence to PIP guidelines among RNs, with attitude emerging as the dominant predictor. Knowledge alone was insufficient, and its effect on bedside adherence was expressed primarily through RNs’ evaluative orientations and habitual clinical behaviors. In addition, although not incorporated in the mediation analysis, the coexistence of comprehensive competency certification in the context of minimal formal PI education and critically low adherence to PIP guidelines highlights a concerning disconnect between credentialing processes and actual clinical competence. Improving PIP adherence necessitates integrated interventions that address all three KAP domains, and competency certification should be aligned with substantive and structured education as a core patient safety strategy for PIP.

## Figures and Tables

**Table 1:** Demographic and Professional Characteristics of Participants (N = 166).

Characteristic	n	%
**Gender**		
Male	29	17.5
Female	137	82.5
**Ethnicity**		
Arab (Saudi and other Arab nationals)	13	7.8
Indian	65	39.2
Filipino	76	45.8
Others	12	7.2
**Educational Qualification**		
Diploma	36	21.7
Bachelor’s degree	98	59.0
Master’s degree	32	19.3
**Clinical Unit**		
Acute care	87	52.4
Critical care	79	47.6
**Previously Received PIP** **Education or Training**		
Yes	11	6.6
No	155	93.4
**Competency in PIP**		
Yes	149	89.8
No	17	10.2
**Last Read PIP Guidelines**		
<1 year ago	96	57.8
1–2 years ago	70	42.2

Note. PIP = pressure injury prevention; acute care = emergency department and medical units; critical care = intensive care units and critical units.

**Table 2:** Descriptive Statistics and Distribution Properties of Continuous Variables Included in the Parallel Mediation Model (N = 166).

Variable	Min	Max	Mean	SD	Skewness	Kurtosis
Knowledge	2	16	7.31	3.6	0.40	−0.60
Attitude	13	36	28.20	5.2	−0.60	0.11
Practice	22	50	36.10	3	−0.13	−0.83
Adherence	18	75	28.40	11.8	1.80	3.60
Age (years)	24	60	36.80	7.7	0.65	0.30
Years of experience	2	48	11.70	5.6	0.30	−0.80

Note. SD = standard deviation.

**Table 3:** Pearson Correlation Matrix for Knowledge, Attitude, Practice, and Adherence to PIP Guidelines (N = 166).

Variable	Knowledge	Attitude	Practice	Adherence
Knowledge	-			
Attitude	0.485 ***	-		
Practice	0.580 ***	0.512 ***	-	
Adherence	0.477 ***	0.505 ***	0.480 ***	-

Note. *** *p* < 0.001 (two-tailed).

**Table 4:** Summary of Multiple Linear Regression Analysis Predicting Overall PIP Adherence.

Predictor	B	SE	β	*t*	*p*	95% CI	VIF
(Constant)	−4.99	5.50		−0.9	0.36	[−15.80, 5.80]	-
Knowledge	0.70	0.20	0.22	3.10	0.003	[0.25, 1.20]	1.7
Attitude	0.70	0.15	0.30	4.40	<0.001	[0.38, 0.98]	1.5
Practice	0.80	0.30	0.20	2.80	0.006	[0.20, 1.40]	1.8
Age	−0.20	0.10	−0.08	−1.60	0.12	[−0.34, 0.04]	1.3
Years of Experience	0.30	0.12	0.12	2.10	0.04	[0.01, 0.50]	1.2

Note. B = unstandardized coefficient; SE = standard error; β = standardized coefficient; CI = confidence interval; VIF = variance inflation factor. *R* = 0.60; *R*^2^ = 0.40; adjusted *R*^2^ = 0.35; *F*(5, 160) = 24.50, *p* < 0.001.

**Table 5:** Parallel Multiple Mediation Analysis: Direct and Indirect Effects of Knowledge on Overall PIP Adherence via Attitude and Practice (N = 166).

Path/Effect	Coeff/Effect	SE	*t*/Boot	LLCI	ULCI
Path a1: Knowledge → Attitude	0.6	0.09	*t* = 6.3	0.40	0.80
Path a2: Knowledge → Practice	0.4	0.05	*t* = 7.9	0.30	0.50
Path b1: Attitude → Adherence	0.7	0.20	*t* = 4.4	0.40	0.98
Path b2: Practice → Adherence	0.8	0.30	*t* = 2.8	0.23	1.40
Direct Effect: Knowledge → Adherence (c’)	0.7	0.23	*t* = 3.1	0.25	1.20
Indirect Effect via Attitude (a1 × b1)	0.4	0.10	Boot	0.20	0.64
Indirect Effect via Practice (a2 × b2)	0.3	0.12	Boot	0.01	0.60
Total Indirect Effect	0.7	0.17	Boot	0.43	1.10

Note. Bootstrap (Boot) confidence intervals (CIs) based on 5000 resamples. Covariates: age, years of experience. LLCI = lower limit confidence interval; ULCI = upper limit confidence interval. Direct and indirect effects are unstandardized. A 95% CI excluding zero indicates a significant effect.

## Data Availability

The data presented in this study are available on request from the corresponding author due to ethical restrictions, as the study setting is a military medical facility.

## Supplementary Materials

The following supporting information can be downloaded at: https://www.mdpi.com/article/10.3390/healthcare14121760/s1. Conceptual framework explaining the mediation model, the checklist of Strengthening the Reporting of Observational Studies in Epidemiology (STROBE) guidelines, and participant flow diagram.

## Abbreviations

The following abbreviations are used in this manuscript.

APuP	Attitude towards Pressure Ulcer Prevention
HAPI	Hospital-acquired Pressure Injury
ICU	Intensive Care Unit
IRB	Institutional Review Board
KAP	Knowledge, Attitude and Practices
PI	Pressure Injury
PIP	Pressure Injury Prevention
PSMMC	Prince Sultan Military Medical City
QARPPU	Questionnaire to evaluate nurses’ Adherence to Recommendations for Preventing Pressure Ulcers
Q–Q plot	Quantile–quantile Plot
RN	Registered Nurse
STROBE	Strengthening the Reporting of Observational Studies in Epidemiology
VIF	Variance Inflation Factor
PUKAT	Pressure Ulcer Knowledge Assessment Tool
