# Supplementary material for: Cross-Sectional Mediation Analysis: How Registered Nurses’ Knowledge, Attitude, and Practice Influence Adherence to Pressure Injury Prevention
Source: Healthcare (Basel). 2026 Jun 18;14(12):1760. doi: 10.3390/healthcare14121760 (PMC13299708; doi:10.3390/healthcare14121760)
Supplement: Supplementary file 1 [file healthcare-14-01760-s001.zip › healthcare-4375097-supplementary.pdf]

Checklist (STROBE Statement)

Manuscript: The Relationship Between Registered Nurses' Knowledge and Adherence to Pressure Injury Prevention: The Mediating Roles of Attitude and Practice in a Cross-sectional Study

Section/Topic	Item No.	Recommendation	Reported	Location in Manuscript
TITLE AND ABSTRACT				
Title & Abstract	1a	Indicate the study's design with a commonly used term in the title or the abstract	Yes	Page 1, lines (1-4)
	1b	Provide in the abstract an informative and balanced summary of what was done and what was found	Yes	Abstract (Page 1): Structured abstract covers Introduction, Methods, Results, and Discussion/Conclusion with key findings, sample, methods, and outcomes reported.
INTRODUCTION				
Background / Rationale	2	Explain the scientific background and rationale for the investigation being reported	Yes	Introduction (Pages 2–3): PI burden, global prevalence (12.8%), economic costs ($26.8B), Saudi ICU rates (>26%), nurses' role in PIP, and KAP as determinants described with citations.
Objectives	3	State specific objectives, including any prespecified hypotheses	Yes	Introduction, final paragraph (Page 3): Study aim and four specific objectives stated: assessing KAP levels; examining knowledge–mediator relationships; evaluating direct effect of knowledge; and determining mediation extent.
METHODS				
Study Design	4	Present key elements of study design early in the paper	Yes	Section 2.1 (Page 4): 'A cross-sectional analytical design utilizing mediation was implemented.' Design rationale and STROBE compliance stated.

Section/Topic	Item No.	Recommendation	Reported	Location in Manuscript
Setting	5	Describe the setting, locations, and relevant dates, including periods of recruitment, exposure, follow-up, and data collection	Yes	Section 2.2 (Page 4): PSMMC, Riyadh, Saudi Arabia; 52 nursing units; recruitment 5–10 March 2026; data collection 5–15 March 2026 (Section 2.7, p. 5).
Participants	6a	Give the eligibility criteria, and the sources and methods of selection of participants	Yes	Section 2.3 (Page 4): Inclusion criteria (direct bedside care, ≥1 year experience); exclusion criteria (administrative/non-clinical roles, absence, students/interns) clearly stated.
Variables	7	Clearly define all outcomes, exposures, predictors, potential confounders, and effect modifiers	Yes	Section 2.6 (Page 4-5): Knowledge (PUKAT 2.0), attitude (APuP), practice (adapted Thomas & Nain), adherence (QARPPU) defined. Age and years of experience identified as covariates. Conceptual framework figure (p. 3) illustrates variable roles.
Data Sources / Measurement	8*	For each variable of interest, give sources of data and details of methods of assessment. Describe comparability of assessment methods if more than one group	Yes	Section 2.6 (Page 4-5): Each instrument described in detail—developer, item count, scale, scoring, and reliability (PUKAT 2.0 ICC=0.69; APuP α=0.79; Practice α=0.89; QARPPU α=0.89; overall α=0.81). Single-group design; comparability not applicable.
Bias	9	Describe any efforts to address potential sources of bias	Yes	Section 2.5 (Page 4): Convenience sampling acknowledged as potentially introducing selection bias; sequential approach to all eligible RNs used to improve representativeness. Self-report/social desirability bias acknowledged in Limitations (p. 16). No specific blinding or validation procedures described to mitigate bias.

Section/Topic	Item No.	Recommendation	Reported	Location in Manuscript
Study Size	10	Explain how the study size was arrived at	Yes	Section 2.4 (Page 4): A priori power analysis via G*Power 3.1.9.7; medium effect size (f^2=0.15), α=0.05, power=0.95, 5 predictors → minimum N=138; inflated to 166 to accommodate 20% non-response rate.
Quantitative Variables	11	Explain how quantitative variables were handled in the analyses. If applicable, describe which groupings were chosen and why	Yes	Section 2.9 (Page 5-6): All continuous variables retained as continuous in regression and mediation analyses. Normality assessed via skewness (±2) and kurtosis (±7) thresholds. No categorisation performed.
Statistical Methods	12a	Describe all statistical methods, including those used to control for confounding	Yes	Section 2.9 (Page 5-6): IBM SPSS v30 and PROCESS macro; descriptive statistics; Pearson correlation; multiple linear regression with KAP + covariates (age, experience); parallel mediation (PROCESS Model 4, 5,000 bootstrap resamples, 95% BC-CI). Collinearity via VIF/Tolerance; Durbin-Watson for autocorrelation.
	12b	Describe any methods used to examine subgroups and interactions	Yes	subgroup nationality group not significant
	12c	Explain how missing data were addressed	Yes	Results (Page 7): 'A total of 166 nurses participated in this study, with no missing value across any variable.' Complete-case analysis used by default.
	12d	If applicable, describe analytical methods taking account of sampling strategy	Yes	Section 2.5 (Page 4): Convenience sampling described. No design-based weighting or corrections applied; authors note this as a limitation.
	12e	Describe any sensitivity analyses	No	No sensitivity analyses reported.

Section/Topic	Item No.	Recommendation	Reported	Location in Manuscript
RESULTS				
Participants	13a*	Report numbers of individuals at each stage of study — numbers potentially eligible, examined for eligibility, confirmed eligible, included, completed follow-up, and analyzed	Yes	Results (Page 7): 166 nurses participated and analyzed. Numbers potentially eligible or approached not explicitly stated. No attrition (all responded completely).
	13b	Give reasons for non-participation at each stage	Yes	Reasons for non-participation not reported. Sample size inflation assumed 20% non-response (Section 2.4) but actual non-participation not documented.
	13c	Consider use of a flow diagram	Yes	Participant flow diagram is included in Page 7.
Descriptive Data	14a*	Give characteristics of study participants (demographic, clinical, social) and information on exposures and potential confounders	Yes	Table 1 (Page 8): Gender, ethnicity, educational qualification, clinical unit, PI information history, competency status, and last guideline reading date provided for all 166 participants.
	14b*	Indicate number of participants with missing data for each variable of interest	Yes	Results (Page 7): Explicitly states no missing values across any variable. Table 2 (Page 9) reports descriptive statistics for all continuous variables.
Outcome Data	15*	Report numbers of outcome events or summary measures	Yes	Table 2 (Page 9): Adherence (primary outcome) mean=28.4, SD=11.8, range 18–75, skewness=1.8, kurtosis=3.6. Summary measures for all study variables reported.
Main Results	16a	Give unadjusted estimates and, if applicable, confounder-adjusted estimates and their precision (95% CI). Make clear which confounders were adjusted for and why included	Yes	Table 4 (Page 10-11): Adjusted regression estimates with B, SE, β, t, p, 95% CI, VIF reported. Table 3 (Page 10): Unadjusted Pearson correlations provided. However, unadjusted (bivariate) regression

Section/Topic	Item No.	Recommendation	Reported	Location in Manuscript
				estimates for each predictor are not separately presented—only correlations serve as unadjusted estimates. Age and experience included as covariates per conceptual framework.
	16b	Report category boundaries when continuous variables were categorized	N/A	No continuous variables were categorized.
	16c	If relevant, consider translating estimates of relative risk into absolute risk for a meaningful time period	N/A	Study examines predictors of adherence using regression; relative/absolute risk translation not applicable.
Other Analyses	17	Report other analyses done — subgroups, interactions, sensitivity analyses	Yes	Pearson correlation matrix (Table 3, Page 10) and regression diagnostics (Durbin-Watson, Q-Q plots, VIF) reported as supplementary analyses. No subgroup, interaction, or sensitivity analyses conducted.
DISCUSSION				
Key Results	18	Summarize key results with reference to study objectives	Yes	Discussion (Pages 12-17): Opens with summary of mediation findings and key regression results, directly referenced to study objectives. Adherence, KAP scores, and mediating pathways all discussed.
Limitations	19	Discuss limitations of the study, taking into account sources of potential bias or imprecision. Discuss both direction and magnitude of any potential bias	Yes	Section 4 (Page 17): Four limitations listed cross-sectional design (causal inference), self-report bias (upper-bound adherence estimate), single-center design, and unmeasured confounders (staffing, resources, culture). Direction of bias noted for self-report (overestimation). Magnitude of bias not quantified.

Section/Topic	Item No.	Recommendation	Reported	Location in Manuscript
Interpretation	20	Give a cautious overall interpretation of results considering objectives, limitations, multiplicity of analyses, results from similar studies, and other relevant evidence	Yes	Discussion (Pages 12-17) and Conclusion (Page 17): Interpretation contextualized with international literature (Belgium, Spain, China, Australia, Malaysia, Ethiopia, Saudi Arabia). Partial mediation model interpreted cautiously. Limitations acknowledged in implications section.
Generalizability	21	Discuss the generalizability (external validity) of the study results	Yes	Limitations (Page 17): 'Single-site cross-sectional evidence informs intervention design rather than justifying immediate system-wide change.' Single-center, predominantly expatriate workforce, and specific institutional context are noted as generalizability constraints.
OTHER INFORMATION				
Funding	22	Give the source of funding and the role of the funders for the present study	Yes	Funding section (Page 18): 'This study did not receive funding.' Acknowledgment credits Deanship of Scientific Research at King Saud University.

* Legend: Yes = Fully reported   Partial = Partially reported   No = Not reported   N/A = Not applicable

Give information separately for exposed and unexposed groups (not applicable in single-group cross-sectional design).

Reference: Vandenbroucke JP, et al. Strengthening the Reporting of Observational Studies in Epidemiology (STROBE): explanation and elaboration. Epidemiology. 2007;18(6):805-35.
